# Plasma Superoxide Dismutase-1 as a Surrogate Marker of Vivax Malaria Severity

**DOI:** 10.1371/journal.pntd.0000650

**Published:** 2010-04-06

**Authors:** Bruno B. Andrade, Antonio Reis-Filho, Sebastião Martins Souza-Neto, Imbroinise Raffaele-Netto, Luis M. A. Camargo, Aldina Barral, Manoel Barral-Netto

**Affiliations:** 1 Centro de Pesquisas Gonçalo Moniz (Fundação Oswaldo Cruz [FIOCRUZ]), Salvador, Brazil; 2 Faculdade de Medicina da Bahia (Universidade Federal da Bahia), Salvador, Brazil; 3 Departamento de Parasitologia, Instituto de Ciências Biológicas, Universidade de São Paulo, São Paulo, Brazil; 4 Faculdade de Medicina, Faculdade São Lucas, Porto Velho, Brazil; 5 Instituto de Investigação em Imunologia, Instituto Nacional de Ciência e Tecnologia (INCT), São Paulo, Brazil; New York University School of Medicine, United States of America

## Abstract

**Background:**

Severe outcomes have been described for both *Plasmodium falciparum* and *P. vivax* infections. The identification of sensitive and reliable markers of disease severity is fundamental to improving patient care. An intense pro-inflammatory response with oxidative stress and production of reactive oxygen species is present in malaria. Inflammatory cytokines such as tumor necrosis factor-alpha (TNF-alpha) and antioxidant agents such as superoxide dismutase-1 (SOD-1) are likely candidate biomarkers for disease severity. Here we tested whether plasma levels of SOD-1 could serve as a biomarker of severe vivax malaria.

**Methodology/Principal Findings:**

Plasma samples were obtained from residents of the Brazilian Amazon with a high risk for *P. vivax* transmission. Malaria diagnosis was made by both microscopy and nested PCR. A total of 219 individuals were enrolled: non-infected volunteers (n = 90) and individuals with vivax malaria: asymptomatic (n = 60), mild (n = 50) and severe infection (n = 19). SOD-1 was directly associated with parasitaemia, plasma creatinine and alanine amino-transaminase levels, while TNF-alpha correlated only with the later enzyme. The predictive power of SOD-1 and TNF-alpha levels was compared. SOD-1 protein levels were more effective at predicting vivax malaria severity than TNF-alpha. For discrimination of mild infection, elevated SOD-1 levels showed greater sensitivity than TNF-alpha (76% vs. 30% respectively; p<0.0001), with higher specificity (100% vs. 97%; p<0.0001). In predicting severe vivax malaria, SOD-1 levels exhibited higher sensitivity than TNF-alpha (80% vs. 56%, respectively; p<0.0001; likelihood ratio: 7.45 vs. 3.14; p<0.0001). Neither SOD-1 nor TNF-alpha could discriminate *P. vivax* infections from those caused by *P. falciparum*.

**Conclusion:**

SOD-1 is a powerful predictor of disease severity in individuals with different clinical presentations of vivax malaria.

## Author Summary

Despite being considered a relatively benign disease, *Plasmodium vivax* infection has been associated with fatal outcomes due to treatment failure or inadequate health care. The identification of sensitive and reliable markers of disease severity is important to improve the quality of patient care. Although not imperative, a good marker should have a close causative relationship with the disease pathogenesis. During acute malaria, an intense inflammatory response and a well-documented oxidative burst are noted. Among the free radicals released, superoxide anions account for the great majority. The present study aimed to evaluate the reliability of using an antioxidant enzyme, responsible for the clearance of superoxide anions, as a marker of vivax malaria severity. Thus, we investigated individuals from an Amazonian region highly endemic for vivax malaria with the goal of predicting infection severity by measuring superoxide dismutase-1 (SOD-1) plasma levels. In addition, we compared the predictive power SOD-1 to that of the tumor necrosis factor (TNF)-alpha. SOD-1 was a more powerful predictor of disease severity than TNF-alpha in individuals with different clinical presentations of vivax malaria. This finding opens up new approaches in the initial screening of severe vivax malaria cases.

## Introduction

Severe malaria presents a relevant public health problem worldwide, affecting the socio-economic development of many communities. The identification of predictors of disease severity is critical to improve patient care. Most of the actual knowledge regarding the immunopathological determinants of malaria severity refers to infection caused by *Plasmodium falciparum*, but growing evidence also associates vivax malaria with severe complications [1], [2]. Together with rising documentation of drug resistance worldwide, the complications of *Plasmodium vivax* infection represents a global health threat. Therefore, identifying markers of disease severity is essential to improve clinical management. Plasma TNF-alpha levels have been described as a biomarker for the estimation of disease severity for *P. falciparum* [3] and is associated with clinical severity in *P. vivax* [4] infections, but there is scarce data evaluating or validating more sensitive and reliable predictors of severe disease.

During malaria infection, reactive oxygen species (mainly superoxide anions) are produced at high levels, inducing parasite killing and tissue damage [5]. To circumvent this biological injury, the anti-oxidant enzyme Cu/Zn superoxide dismutase (SOD-1) converts these unstable free radicals into hydrogen peroxide (H_2_O_2_), which can be removed by the catalase and glutathione systems [6]. Studies in both mice [7] and humans [8] have correlated the SOD-1 activity with tissue damage. Therefore, investigating markers related to oxidative stress could provide useful tools to manage malaria. The present work shows that the plasma level of SOD-1 is a surrogate marker of severe vivax malaria in a population from the Brazilian Amazon, in which *P. vivax* infection is highly endemic. The performance of SOD-1 as a predictor of disease severity even surpasses that of TNF-alpha.

## Methods

### Objective

The objective of this study was to test whether the plasma level of SOD-1, an antioxidant enzyme, could predict vivax malaria severity with equivalent of better efficacy compared to the currently used marker TNF-alpha.

### Study design and participants

Plasma samples were obtained from individuals living in Buritis, a recently urbanized municipality in Rondônia, Brazilian Amazon, with a high risk for vivax malaria transmission [9], during June 2006 and August 2007. Active and passive malaria case detections were performed. These included home visits and study of individuals who sought care at the diagnostic center of Brazilian National Foundation of Health (FUNASA). In addition, patients admitted to the Buritis municipal Hospital with clinical signs of mild or severe malaria [10] were also asked to participate in the study. All individuals from fifteen to seventy years, of both sexes, who had been living in the endemic area for more than six months, were invited to be included in the study. The malaria diagnosis was performed using two methods (double-blinded). First, patients were screened by thick smear examination using field microscopy and the parasitaemia (parasites/uL) was quantified in positive cases. Further, nested PCR was performed in all whole blood samples to confirm the diagnosis. Exclusion criteria were viral hepatitis (A, B, C, and D), chronic alcoholism, human immunodeficiency virus type 1 infection, yellow fever, leptospirosis, cancer and chronic degenerative diseases, sickle cell trait and the use of hepatotoxic or immunosuppressant drugs. Two individuals presenting *P. malariae* infection were identified and excluded from the study. In addition, 16 age-matched people infected with *P. falciparum* (uncomplicated forms) were invited to participate. In the last phase of the study, plasma samples from these individuals with *P. falciparum* malaria were used in order to assess if the markers compared were useful to discriminate *P. vivax* from *P. falciparum* infections.

After obtaining the parasitological diagnosis, all vivax malaria positive cases were followed for 30 days. Individuals infected with *P. falciparum* were not included in the follow up. Infected individuals who remained without any presumptive malaria symptoms were considered asymptomatic; patients presenting clinical or laboratory signs of complicated malaria [10] were considered severe cases, while those who were symptomatic without any complication were mild cases. In hospitalized participants presenting with severe disease, two plasma samples were obtained: one at the hospital admission and other seven days after malaria treatment initiation. Thus, of 415 individuals initially approached, 58 were excluded for meeting exclusion criteria, 86 withdrawn consent and 36 neglected the follow up. The sample was then composed of non-infected volunteers (n = 90) and individuals with different clinical presentations of vivax malaria: asymptomatic (n = 60), mild (n = 50) and severe infection (n = 19). The detailed clinical descriptions of the participants together with the outcomes have been already addressed by our group [11]. A summary of the baseline characteristics of the participants is illustrated in Table 1. All the malaria cases were treated by the FUNASA health care professionals according to the FUNASA standardized protocols. The flow chart of the validation study is shown in Figure S1.

**Table 1:** Baseline characteristics of the participants.

Variables	*Plasmodium vivax* infection			
	Non-infected	Asymptomatic	Mild	Severe
	N = 90	N = 60	N = 50	N = 19
**Male – no. (%)**	39 (43.3)	30 (50.0)	22 (44.0)	10 (52.6)
**Age – year** *				
Median	38.0	42.0	33.0	22.0
Interquartile interval	25.0–51.0	32.0–48.2	26.7–48.0	16.0–35.0
**Parasitaemia (parasites/uL)** *				
Median	0	73	4,798	49,358
Interquartile interval	0	54.0–85.0	2,934–7,483	32,796–54,244
**Haemoglobin (g/dL)** *				
Median	13.2	11.5	8.9	6.4
Interquartile interval	9.2–14.5	9.5–14.2	7.3–12.6	5.8–7.4
**Serum creatinine (mg/dL)** *				
Median	0.85	0.9	1.1	1.7
Interquartile interval	0.7–1.2	0.7–1.2	0.7–1.3	1.42–2.45
**ALT (U/L)** *				
Median	42.35	40	58.3	238.4
Interquartile interval	37.28–53.58	23.25–65.78	43.6–87.5	105.5–364.6
UNL	1.06	1	1.46	4.96
**Clinical presentation – no. (%)** § *				
Splenomegaly	-	-	8 (16.0)	6 (31.6)
Hypotension	-	-	6 (12.0)	14 (73.68)
Jaundice	-	-	9 (18.0)	7 (36.8)

ALT: alanine amino-transferase. UNL: Upper normal levels. Data represent the number of times the median of ALT is higher than the standardized normal laboratory level (40U/L). Ordinal variables were compared using the Kruskal Wallis test with Dunn's multiple comparisons. The prevalence of male gender was compared between the groups using chi-square test.

*Differences were significant between groups (P<0.05).

§The groups were compared using chi-square test.

### Ethics statement

Written informed consent was obtained from all participants, and all clinical investigations were conducted according to the principles expressed in the Declaration of Helsinki. The project was approved by the institutional review board of the Faculdade de Medicina, Faculdade São Lucas, Rondônia, Brazil, where the study was performed.

### Nested PCR for malaria diagnosis

The molecular diagnosis of malaria was performed using nested PCR, as described previously [12]. Briefly, 300 µL of whole blood collected on EDTA was prepared for DNA extraction through the phenol-chloroform method followed by precipitation with sodium acetate and ethanol. The first PCR rDNA amplification was performed with *Plasmodium* genus-specific primers named PLU5 and PLU6. Positive samples yielded a 1,200-bp fragment, which served as template for the nested reaction. The nested PCR amplification was performed with species-specific primers for 30 cycles at annealing temperatures of 58°C for *P. falciparum* (Fal1 and Fal2 primers), and 65°C for *P. vivax* (Viv1 and Viv2 primers) or *P. malariae* (Mal1 and Mal2 primers). The fragments obtained for *P. vivax* were of 120 bp, whereas for *P. falciparum* and *P. malariae* were 205 bp and 144 bp, respectively. The oligonucleotide sequences of each primer used are listed in Table 2. The products were visualized in 2% agarose gel stained with ethidium bromide. One uninfected blood sample was included for every twelve samples processed to control for cross-contamination. Fifteen percent of positive PCR samples were re-tested to confirm the amplification of plasmodial DNA. All tests were performed and confirmed at our main laboratory at the Centro de Pesquisas Gonçalo Moniz, Brazil.

**Table 2:** Primers used in Nested PCR reactions.

Primer	Oligonucleotide Sequence 5′-3′	Base Pairs
PLU5	CCTGTTGTTGCCTTAAACTTC	1,200
PLU6	TTAAAATTGTTGCAGTTAAAA	
Fal1	TTAAACTGGTTTGGGAAAACCAAATATATT	205
Fal2	ACACAATGAACTCAATCATGACTACCCGTC	
Viv1	CGCTTCTAGCTTAATCCACATAACTGATAC	120
Viv2	ACTTCCAAGCCGAAGCAAAGAAAGTCCTTA	
Mal1	ATAACATAGTTGTACGTTAAGAATAACCGC	144
Mal2	AAAATTCCCATGCATAAAAAATTATACAAA	

PLU: *Plasmodium sp*, Fal: *Plasmodium falciparum*, Viv: *Plasmodium vivax*, Mal: *Plasmodium malariae*.

### Plasma cytokine measurements

Plasma levels of TNF-alpha were measured using the Cytometric Bead Array - CBA® (BD Biosciences Pharmingen, USA) according to the manufacturer's protocol, with all samples run in a single assay. The flow cytometric assay was performed and analyzed by a single operator, and standard curves were derived from cytokine standards. The minimum limit of detection was 3.7 pg/mL.

### Laboratory assessment of organ dysfunction

Plasma measurements of creatinine, alanine amino-transaminase (ALT) and haemoglobin were made at the clinical laboratory of Faculdade São Lucas and at the Laboratório LPC (Salvador, Bahia. Brazil).

### Plasma superoxide dismutase measurements

SOD-1 plasma concentrations were measured using the Cu/Zn Superoxide Dismutase ELISA Kit according to the manufacturer's protocol (Calbiochem, EMD chemicals, Darmstadt, Germany). Briefly, human serum was diluted 1∶200 in PBS and distributed in a sensitized 96-wells plate. The samples were incubated for one hour at room temperature with HRP-conjugated anti-Cu/Zn SOD antibody. A colorimetric substrate was added for ten minutes, being the system protected from intense light. The reaction was stop and the plate read at 450nm. The SOD activity assay was performed using the Superoxide Dismutase Colorimetric Assay Kit according to the manufacturer's protocol (Cayman chemical, Ann Arbor, MI, USA). Briefly, radical detector was added to a sensitized 96-wells plate. Pre-diluted (1∶50) samples were distributed in wells. The reaction was started using Xanthine Oxidase, and the plate was read after twenty minutes at 450nm. One unit of SOD is defined as the amount of enzyme needed to exhibit 50% dismutation of the superoxide radical.

### Statistical analysis

The Kruskal-Wallis test with Dunn's multiple comparisons or linear trend analysis was used to compare SOD-1 and TNF-alpha levels according to different clinical presentations of vivax malaria infection. The Mann-Whitney test was used to verify differences between asymptomatic and symptomatic, between mild and severe vivax malaria or between *P. vivax* and *P. falciparum* infections. Correlations between SOD-1 or TNF-alpha levels and severity factors were performed using the Spearman test. Receiver-operator characteristic (ROC) curves with C-statistics were used to establish the threshold value of SOD-1 and TNF-alpha able to discriminate between mild and severe infection. A p value<0.05 was considered statistically significant.

## Results

**Figure 1:**
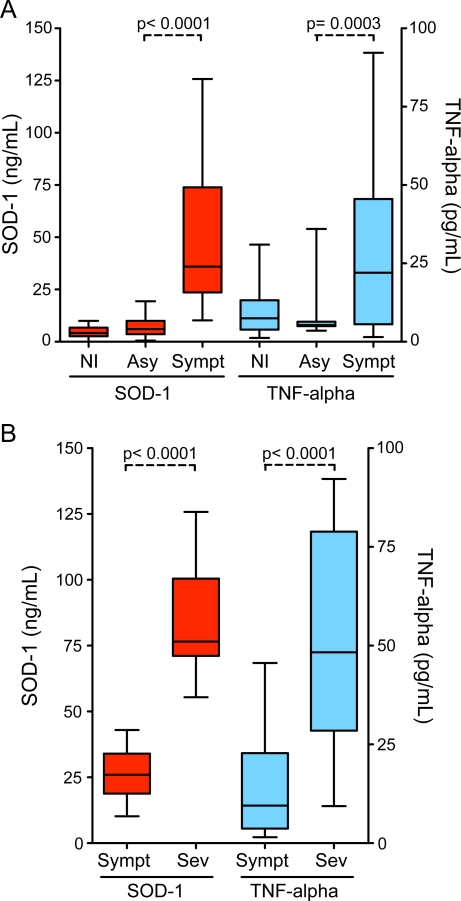
Plasma SOD-1 and TNF-alpha as markers of severe vivax malaria. A, SOD-1 protein and TNF-alpha plasma levels according to vivax malaria clinical severity. NI, non-infected volunteers (n = 90); Asy, asymptomatic infection (n = 60); Sympt, symptomatic infection (n = 69). Differences among the groups were calculated using the Kruskal Wallis analysis of variance with Dunn's multiple comparisons test. B, Plasma levels of SOD-1 and TNF-alpha in individuals with mild *P. vivax* infection (n = 50) compared to those with severe vivax malaria (Sev; n = 19). Boxes represent median and interquartile interval; whiskers represent maximum and minimum values. Differences were estimated using Mann-Whitney test. Lines represent median values. P values are shown in each graph.

**Figure 2:**
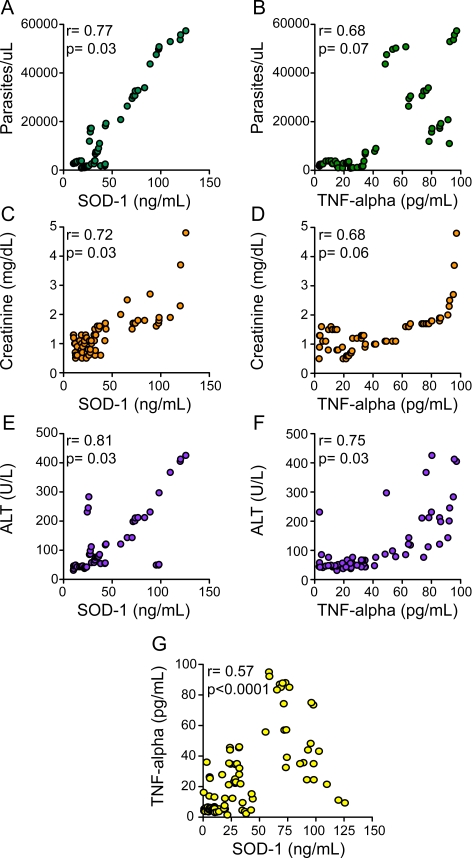
Correlations between plasma SOD-1 or TNF-alpha concentrations and laboratory parameters of malaria severity. Correlation of SOD-1 or TNF-alpha with several laboratory parameters in symptomatic vivax malaria patients (n = 69). Column at left (A, C and E): Correlations of SOD-1 with parasitaemia (A), plasma creatinine (C) and alanine amino-transaminase (ALT; E). Column at right: Correlations of TNF-alpha to parasitaemia (B), plasma creatinine (D) and ALT (F). Correlation between TNF-alpha and SOD-1 plasma protein levels is shown in G. The statistical significances were calculated using the Spearman test. The values of p and r are illustrated in each graph.

**Figure 3:**
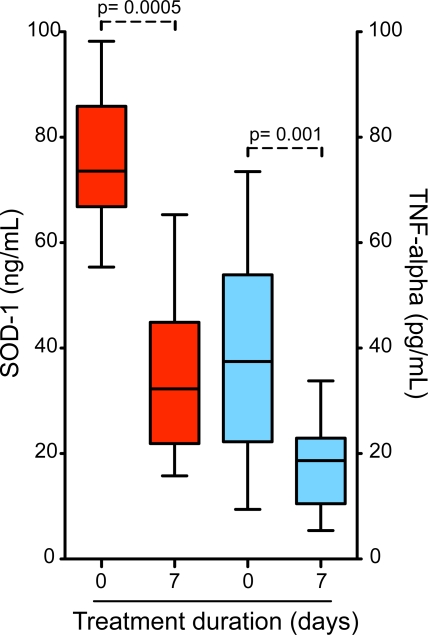
Effect of anti-malaria treatment on plasma concentrations of SOD-1 and TNF-alpha in individuals with severe vivax malaria. Plasma levels of SOD-1 protein (red boxes, left Y axis) and TNF-alpha (blue boxes, right Y axis) were estimated before treatment (at admission to the Hospital) and after seven days of in-hospital treatment with intravenous quinine and hemodynamic support in individuals with severe vivax infection who successful recovered (n = 13). Boxes represent median and interquartile interval; whiskers represent maximum and minimum values. Wilcoxon matched pairs test was performed to calculate the statistical significance. P values are plotted in each graph.

**Figure 4:**
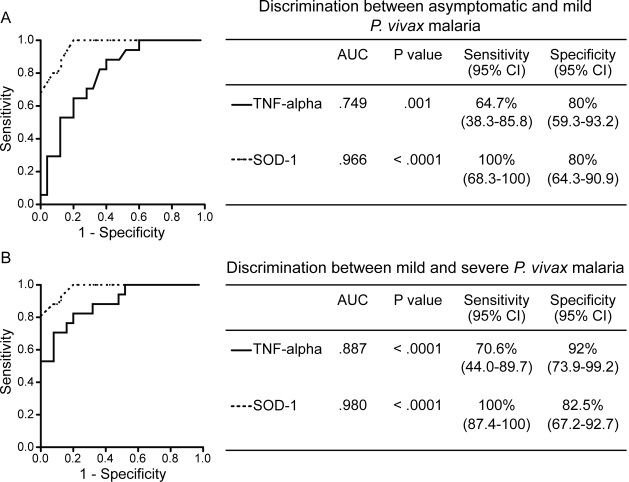
Effectiveness of plasma concentrations of SOD-1 and TNF-alpha measurements as markers of vivax malaria severity. A, ROC curves of SOD-1 (dashed line) and TNF-alpha (solid line) plasma levels for discriminating asymptomatic infection from mild *P. vivax* malaria cases. B, ROC curves of SOD-1 (dashed line) and TNF-alpha levels (solid line) for discriminating severe from mild *P. vivax* malaria cases. C-statistics are illustrated in the tables and were used to verify the validation of the ROC curves and the predictive power of each biomarker. AUC, area under the curves; CI, confidence interval.

**Figure 5:**
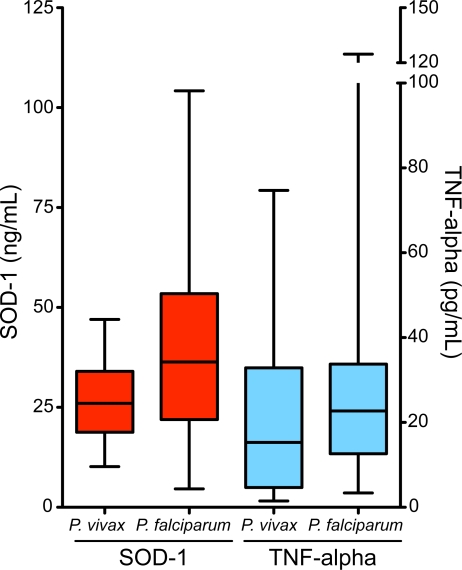
Plasma concentrations of SOD-1 and TNF-alpha during P. vivax and P. falciparum infections. Plasma levels of SOD-1 (red boxes) and TNF-alpha (blue boxes) were measured in patients with mild *P. vivax* (n = 50) or mild *P. falciparum* (n = 16) malaria. Boxes represent median and interquartile interval; whiskers represent maximum and minimum values. The differences between *P. vivax* and *P. falciparum* infections were not significant when compared by the Mann-Whitney test (p>0.05).

Increased vivax malaria severity was associated with higher plasma levels of SOD-1 (P<0.0001; Figure 1A and 1B), with similar trend being noted with regard to SOD activity (P<0.01 for linear trend; data not shown). Considering individuals with mild and severe infections together (n = 69), increased SOD-1 protein levels were correlated with higher parasitaemia (r = 0.77, p = 0.03; Figure 2A), while this correlation did not reach significance for TNF-alpha (r = 0.68, p = 0.07; Figure 2B). In addition, splenomegaly and hypotension were more prevalent in patients with high SOD-1 and TNF-alpha levels compared to those with low levels of both factors (43.2% vs. 5.1% respectively; Fisher's test p = 0.02). Correlation between SOD-1 protein levels and plasma creatinine measurements was r = 0.72 (p = 0.03; Figure 2C), while the correlation between TNF-alpha and creatinine did not achieved statistical significance in the cohort under investigation in this study (r = 0.68, p = 0.06; Figure 2D). Furthermore, a similar pattern was observed regarding the correlation of ALT with SOD-1 protein levels (r = 0.81, p = 0.03; Figure 2E) or TNF-alpha (r = 0. 75, p = 0.03; Figure 2F). SOD-1 protein levels were also directly associated with systemic TNF-alpha (r = 0.57, p<0.0001; Figure 2G). All individuals with severe disease presented with anemia at the time of hospitalization (haemoglobin mean: 6.2±1.4), while in those with mild infection, only 14/50 were anemic (haemoglobin mean: 12.5±2.0). In agreement with previous findings on *P. falciparum* [3] and *P. vivax* [4] infections, individuals with severe malaria displayed higher plasma levels of TNF-alpha than those with asymptomatic parasitaemia or mild disease (Figure 1B). Within the individuals presenting with severe disease who successfully recovered after in-hospital care (n = 13), the systemic levels of both SOD-1 and TNF-alpha decreased at least two fold during the seventh day of anti-malarial treatment (p = 0.0005 and p = 0.001, respectively; Figure 3). We also assessed the possibility of estimating threshold levels of TNF-alpha and SOD-1 to discriminate between asymptomatic and symptomatic infection. As expected, individuals with symptomatic infection (mild or severe) presented higher levels of both TNF-alpha and SOD-1 than those who were symptomless (Figure 1A). SOD-1, however, was a better marker than TNF-alpha (Figure 4A). Moreover, TNF-alpha and SOD-1 levels were elevated in individuals with severe disease compared to mild disease (Figure 1B). SOD-1 was also more powerful than TNF-alpha in predicting severe disease (Figure 4B). In an attempt to address if the plasma levels of both SOD-1 and TNF-alpha were useful to discriminate *P. vivax* from *P. falciparum* malaria, we compared plasma samples from individuals presenting with mild symptomatic vivax malaria and age matched individuals with symptomatic *P. falciparum* infection. Neither SOD-1 nor TNF-alpha could differentiate between the infections (Figure 5).

## Discussion

This study is the first to examine the use of plasma SOD-1 levels as a surrogate marker of *P. vivax* malaria severity. SOD-1 is an important participant in the oxidative stress responses [6]. It has been implicated in several other diseases and infections [13]–[17], and its plasma levels could be a sensitive indicator of inflammatory processes. More recently, SOD-1 has been found to play a deleterious role in cutaneous leishmaniasis, as the interferon-beta inhibition of leishmanicidal activity was mimicked by SOD-1 and antagonized by either pharmacological or small interfering RNA-mediated inhibition of SOD-1 [18]. SOD-1 levels were much more effective in predicting vivax malaria severity than TNF-alpha, a major cytokine related to malaria clinical severity in *P. vivax* infections [19]. For discrimination of mild infection, the use of SOD-1 improves the correct case detection by more than 45% compared with the use of TNF-alpha, in addition to being a better identifier of negative cases. Furthermore, SOD-1 has a higher sensitivity than TNF-alpha in predicting severe vivax malaria, indicating also a higher likelihood ratio to discriminate this clinical condition. This suggests that SOD-1 can serve as an additional and innovative tool in the clinical approach to *P. vivax* malaria cases. The measurements of both SOD-1 and TNF-alpha in the plasma samples are performed using simple ELISA-based kits. It is possible then that costs may be similar depending on the demand. Measuring SOD-1 levels could be used in two situations: (i) identification of patients with severe disease before the development of fatal outcomes and (ii) monitoring the success of therapy and clinical recovery. The viability of applying this methodology in the clinical practice will depend on its priority status in a diagnostic algorithm.

Whether this anti-oxidant enzyme could be used as a marker of disease severity in *P. falciparum* infections was not evaluated here, and should be tested in future investigations. However, in individuals presenting with mild disease, plasma SOD-1 levels could not differentiate between *P. vivax* and *P. falciparum* infections. This suggests that these two parasites may share more similar pathogenetic mechanisms than previously realized.

SOD-1 represents an important defense against oxidative stress within a cell [16], [17]. Furthermore, superoxide radicals are the main ROS produced during acute malaria [5]. The role of SOD-1 in vivax malaria could be either protective or deleterious with regard to the infection outcome. SOD-1 levels may be a reflection of an active injury mechanism or, alternatively, may indicate a counter-regulatory response to the generation of superoxide radicals. Supplementation of SOD-1 protects endothelial cells against the *P. falciparum*-induced oxidative response and apoptosis *in vitro* [19]. Nevertheless, during experimental malaria, mice overexpressing SOD-1 develop oxidative injury associated with increased vulnerability to *P. berghei* [7]. Patients with acute non-complicated *P. falciparum* or *P. vivax* malaria have less catalase activity then non-infected individuals but higher SOD activity [8]. Reduced catalase activity together with increased SOD activity may result in the accumulation of H_2_O_2_, the release of hydroxyl radicals and increased tissue damage during severe malaria.

Although investigations analyzing more patients with broader clinical outcomes are necessary, SOD-1 plasma protein levels seems to represent a useful marker in predicting vivax malaria severity based on the oxidative response status.

### Limitations

This study illustrates the possibility of using SOD-1 levels as a severity biomarker in human *P. vivax* malaria and highlights the likelihood of exploring the future use of the plasma SOD-1 levels as an effective marker of malaria severity. To validate our results, studies investigating samples from different endemic areas are crucial, as local health conditions such as co-infections may limit the effective use of a biomarker. A possible advantage of measuring SOD-1 levels as part of the clinical management in endemic areas cannot be assumed from our results. The use of the SOD-1 as a reliable marker also depends on future field interventions in which the pre-test prediction and cost-effectiveness should be considered. In addition, the specific role of SOD-1 in the immunopathogenesis of severe vivax malaria was not explored in this study and is still being addressed by our group.

## Supporting Information

Checklist S1 STARD checklist.
(0.08 MB PDF)

Figure S1 STARD flowchart.
(0.02 MB PDF)
