# Supplementary figures and images for: Plasma Superoxide Dismutase-1 as a Surrogate Marker of Vivax Malaria Severity
Source: PLoS Negl Trop Dis. 2010 Apr 6;4(4):e650. doi: 10.1371/journal.pntd.0000650 (PMC2850307; doi:10.1371/journal.pntd.0000650)

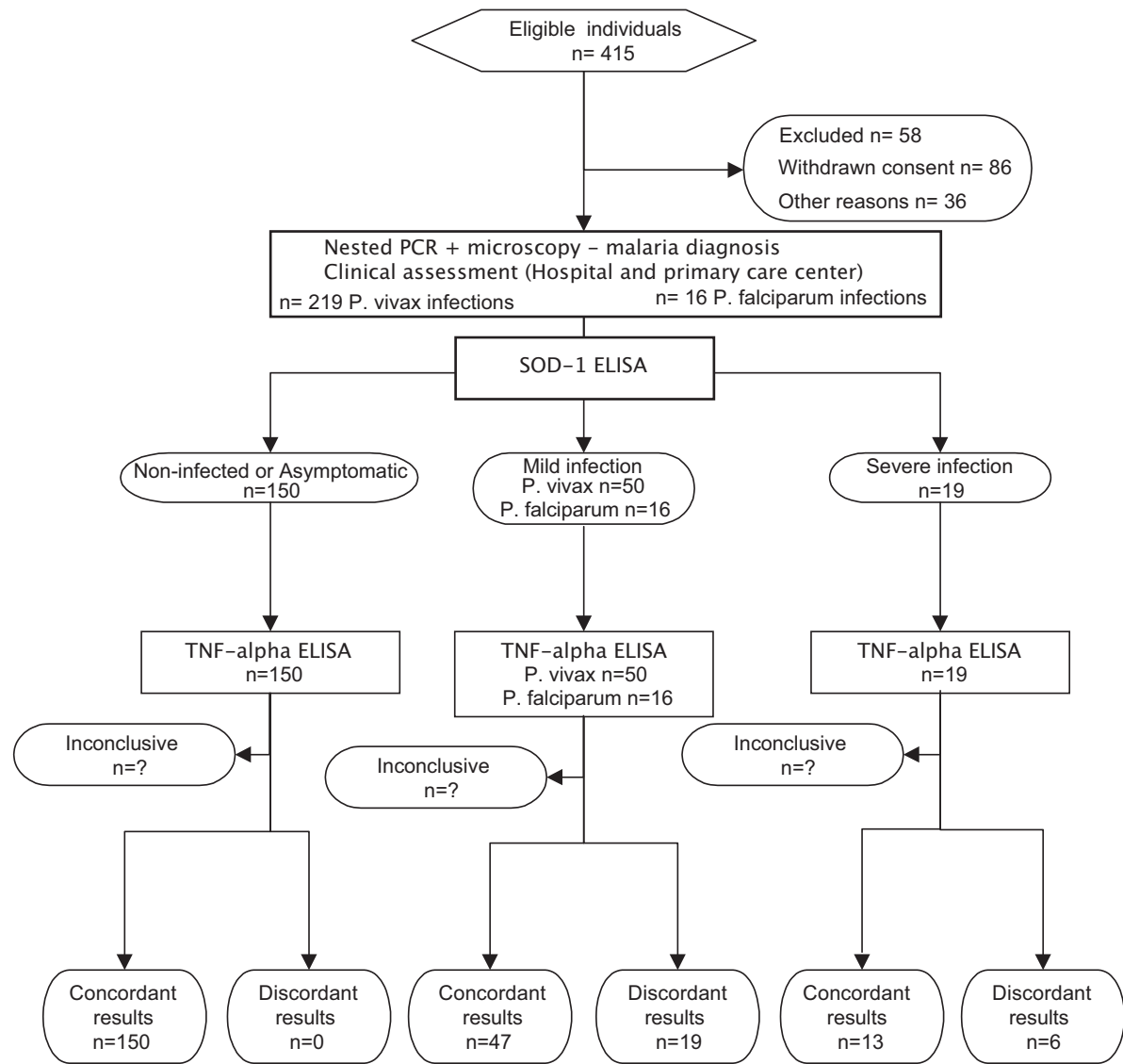

Supplement: Figure S1 — STARD flowchart. (0.02 MB PDF) [file pntd.0000650.s002.pdf]
